# How can we promote mentored student research in Georgia?

**DOI:** 10.21633/jgpha.6.2s02

**Published:** 2016

**Authors:** Nancy C. Webb, Selina A. Smith

**Affiliations:** 1 Institute of Public and Preventive Health, Augusta, GA; 2 Department of Pediatrics, Medical College of Georgia, Augusta, GA; 3 Journal of the Georgia Public Health Association; 4 Department of Family Medicine, Medical College of Georgia, Augusta University, Augusta, GA

## INTRODUCTION

In recent years, there has been a growing momentum to establish healthier environments within communities. For example, the American Recovery and Reinvestment Act of 2009 led to the initiative, Communities Putting Prevention to Work, which provided funding for addressing health inequities (IOM, 2012). One year later, the Patient Protection and Affordable Care Act (ACA) of 2010 was passed with the goal of improving access to health care for all Americans. Private organizations, such as the Robert Wood Johnson Foundation and the Kellogg Foundation, have also made efforts to bring attention to health disparities and improve health for all Americans (IOM, 2012). In spite of these efforts, large-scale disparities persist and are increasing (Rimer, 2016). To diminish morbidity, mortality, and health care costs, it is essential to invest in the health of communities (Rimer, 2016).

Although governmental agencies and private foundations have made progress through targeted public health funding, public health professionals have promoted scholarship to address health inequities through mentored research experiences for students (Hager et al., 2016; Ognibene et al., 2016; Rimer, 2016; Takenoshita et al., 2016). For example, in response to the national focus on health disparities and public health scholarship, the Institute of Public and Preventive Health (IPPH) at Augusta University (AU) established a Summer Public Health Scholars Program (SPHSP) to provide mentored research for students interested in public health. The primary purpose of the program is to foster training in methodology, theory, and practice to enhance understanding of the causes of health disparities, disease prevention, and vulnerability; the secondary purpose is, through immersion experiences, to establish a pipeline of students from various disciplines who are interested in public health.

The goals are four-fold:

1. Supplement student training in public health research methods
2. Foster a career-long commitment to addressing health disparities
3. Promote understanding of career opportunities in public health
4. Provide hands-on research experiences (e.g., reviewing of public health issues; enhancing scientific writing skills; and developing scientific manuscripts, posters, and oral presentations)

In this commentary, we consider lessons learned as we developed and implemented the SPHSP.

## INEQUITIES AND DISPARITIES IN GEORGIA

Public health is the science of protecting and improving communities through education, advancement of healthy lifestyles, and research related to disease and injury prevention (American Public Health Association). In Georgia, social, educational, economic, and health inequities continue to grow among the underserved, rural, minority, and low-income populations (Georgia Department of Public Health, n.d.). These inequities are preventable, needless, and unfair (OHE https://dph.georgia.gov/Health-Equity; WHO http://www.who.int/hia/about/glos/en/index1.html).

Georgia is the ninth most populated state in the US. It has 159 counties, 108 of which are rural. Of the rural counties, 37 are in the Appalachian region (Kaiser Family Foundation, 2014). Residents living in Appalachia, as with residents in other rural areas, are more likely to report poorer health, including diabetes, stroke, and heart disease (ATRN, n.d.). The Rural Health Information Hub (https://www.ruralhealthinfo.org/states/georgia) reports the following statistics:

- The poverty rate in rural Georgia is 24%; non-rural areas of the state have a rate of 17.2%.
- Of those living in rural areas in Georgia, 21% have not completed high school. This compares to 13.6% of the non-rural population.
- The unemployment rate in rural Georgia is 6.6%; in urban Georgia, it is 5.7%.

Georgia falls below national averages in state population health, ranking 38^th^ of the 50 states (Kaiser Family Foundation, 2014). Further, Georgia has not implemented the Medicaid expansion, which means that many low-income adults are uninsured (Kaiser Family Foundation, 2014). There is, however, in Georgia, a concerted effort to eliminate health disparities. For example, the Office of Health Inequity (OHI) of the Georgia Department of Public Health states as its mission: “To lead in the elimination of health disparities and promote a healthy quality of life for all Georgians” (https://dph.georgia.gov/Health-Equity). In developing mentored research programs for scholars, the IPPH exists in a dynamic relationship with the OHI in Georgia’s Department of Public Health.

## PUBLIC HEALTH: DIVERSE IN DISCIPLINES AND DIVERSE IN ITS REACH

The disciplines comprising the public health sector are diverse, including behavioral sciences, environmental health, medicine, and infection prevention. The IPPH has faculty members who conduct public health-related research on cancer prevention and control, correctional health, early childhood education, nutrition, and substance abuse. As an academic research unit, we are in a position to offer structured and mentored research opportunities to scholars. We have a racial and ethnically diverse faculty, representative of the diverse student population at AU. Underrepresented minorities are in a distinctive position to address health disparities research, since they may have a better understanding of barriers to participation in research and other programs (Sopher et al., 2015). Ethnic and racial minorities are underrepresented in the field of public health (Prunuske et al., 2016; Sopher et al., 2015). Over the past two years, the SPHSP has identified and trained scholars who represent diversity in race and ethnicity.

## PREPARING STUDENTS TO PERFORM PUBLIC HEALTH RESEARCH

Typical experiences offered to post-baccalaureate public health students include internships, capstone projects, and thesis/dissertation preparation. The SPHSP model promotes immersion in a 40-hour per week, 10-week period during the summer. Each student accepted into the program is paired, in a one-on-one model, with a mentor faculty member in the IPPH.

Since 2014, post-baccalaureate students from the Medical College of Georgia, the Master of Psychology Program, College of Science and Mathematics, and the Master of Public Health Program, College of Allied Health Sciences have enrolled in the program. By engaging scholars from public health and related disciplines, cross training was promoted within the health sciences. The scholars worked in a central room, which fostered integration of the respective fields and enhanced understanding of the impact of health disparities.

## STUDENT-MENTOR RELATIONSHIPS

The IPPH faculty members possess appropriate characteristics to be effective mentors. These qualities include enthusiasm for research and capacity to model confidence and to share our values and passion (Lefkowitz, 2015). As Lefkowitz (2015) states, the word “enthusiasm” literally means “a god within.” Modeling enthusiasm and optimism is an inspiring characteristic driven by intrinsic motivation. If students are to become prepared to participate in public health scholarship, they need mentors who model a strong focus on health threats and risks (Rimer, 2016). They also need and are willing to receive constructive feedback, to make adequate time commitments, to communicate clearly (Prunuske et al., 2016), to provide socio-emotional support, and to engage in culturally relevant dialogue through inspiring exchanges (Haeger et al., 2016). The quality of mentorship influences the development of both research and academic abilities (Haeger, 2016); productive mentoring relationships can also emerge when the mentor and mentee are similar in their cultural, socioeconomic, and ethnic backgrounds (Haeger et al., 2016).

The SPHSP curriculum served as the foundation for mentor-student relationships and included research guided by a faculty mentor. It also involved a weekly seminar program with featured speakers and discussions about topics related to public health. Each faculty mentor developed a contractual agreement with his or her summer scholar. The agreement addressed the time commitments expected of the faculty member and the scholars, which can be substantial for faculty members who also teach, write, obtain funding, and engage in service opportunities as part of their academic responsibilities. If we are to train the next generation of public health teachers, researchers, and practitioners, we have to commit adequate time. Two end products of the agreement included the development of a poster and a manuscript. On these, scholars served as the first authors, with the mentor serving as the senior author.

## MEASURING SUCCESS

During the past two years, the IPPH has prepared two cohorts of students from diverse educational and ethnic backgrounds to engage in evidence-based research in public health. The scholars learned to identify public health questions, conduct research to answer the questions, develop and present a scientific poster, and submit a manuscript to a peer-reviewed public health journal. Some scholars have also presented posters at state and national conferences. One scholar wrote in her program evaluation, *“ . . .I feel I am more equipped in navigating resources pertinent for completing a comprehensive literature review as well as writing in a manner that is scientifically proficient.”* Takenoshita et al. (2016) noted that having a research mentor is associated with publishing in peer-reviewed journals and that having a mentor prepares the scholar to obtain grants and complete a thesis. These three factors (i.e., publishing, obtaining grants, and completing a thesis) are associated with successful careers. It behooves us to develop, over the next several years, a longitudinal study to follow the careers of our scholar cohorts.

Another indicator of SPHSP success is in the scholars themselves. Both SPHSP cohorts were diverse in ethnicity, socioeconomic status, and training. Mentoring scholars of color and other diverse characteristics is expected to result in a more diversified public health research workforce in Georgia. According to Fuchs et al. (2016, p. S249), “. . . to diversify the future biomedical research workforce attention also must be paid to the opportunities afforded to those at earlier stages of training.” We are pleased to be a part of this movement and feel that the SPHSP model is an effective approach for promoting mentored research by students.
